# Uncovering the mechanism of resveratrol in the treatment of diabetic kidney disease based on network pharmacology, molecular docking, and experimental validation

**DOI:** 10.1186/s12967-023-04233-0

**Published:** 2023-06-12

**Authors:** Shengnan Chen, Bo Li, Lei Chen, Hongli Jiang

**Affiliations:** 1 Department of Critical Care Nephrology and Blood Purification, The First Affiliated Hospital of Xi’an Jiaotong University, West Yanta Road No.277, Xi’an, 710061 Shaanxi China; 2 Department of Nephrology, Ningxia Medical University Affiliated People’s Hospital of Autonomous Region of Ningxia, Yinchuan, 750002 Ningxia China

**Keywords:** Resveratrol, Diabetic kidney disease, Network pharmacology, Molecular docking, Experimental validation

## Abstract

**Background:**

Diabetic kidney disease (DKD) has been the leading cause of chronic kidney disease in developed countries. Evidence of the benefits of resveratrol (RES) for the treatment of DKD is accumulating. However, comprehensive therapeutic targets and underlying mechanisms through which RES exerts its effects against DKD are limited.

**Methods:**

Drug targets of RES were obtained from Drugbank and SwissTargetPrediction Databases. Disease targets of DKD were obtained from DisGeNET, Genecards, and Therapeutic Target Database. Therapeutic targets for RES against DKD were identified by intersecting the drug targets and disease targets. GO functional enrichment analysis, KEGG pathway analysis, and disease association analysis were performed using the DAVID database and visualized by Cytoscape software. Molecular docking validation of the binding capacity between RES and targets was performed by UCSF Chimera software and SwissDock webserver. The high glucose (HG)-induced podocyte injury model, RT-qPCR, and western blot were used to verify the reliability of the effects of RES on target proteins.

**Results:**

After the intersection of the 86 drug targets and 566 disease targets, 25 therapeutic targets for RES against DKD were obtained. And the target proteins were classified into 6 functional categories. A total of 11 cellular components terms and 27 diseases, and the top 20 enriched biological processes, molecular functions, and KEGG pathways potentially involved in the RES action against DKD were recorded. Molecular docking studies showed that RES had a strong binding affinity toward PPARA, ESR1, SLC2A1, SHBG, AR, AKR1B1, PPARG, IGF1R, RELA, PIK3CA, MMP9, AKT1, INSR, MMP2, TTR, and CYP2C9 domains. The HG-induced podocyte injury model was successfully constructed and validated by RT-qPCR and western blot. RES treatment was able to reverse the abnormal gene expression of PPARA, SHBG, AKR1B1, PPARG, IGF1R, MMP9, AKT1, and INSR.

**Conclusions:**

RES may target PPARA, SHBG, AKR1B1, PPARG, IGF1R, MMP9, AKT1, and INSR domains to act as a therapeutic agent for DKD. These findings comprehensively reveal the potential therapeutic targets for RES against DKD and provide theoretical bases for the clinical application of RES in the treatment of DKD.

## Background

The prevalence of diabetes has been increasing at an alarming rate worldwide. According to reports from the International Diabetes Federation, it is estimated that 537 million adults aged 20–79 years were living with diabetes in 2021 [1]. This number is expected to rise to 643 million by 2030 and to 783 million by 2045 [1]. The prevalence of diabetic kidney disease (DKD) is dramatically increasing in parallel with the incidence of diabetes. Recently, DKD has become the leading cause of chronic kidney disease and renal failure in most developed countries [2, 3]. Hyperglycemia is the major driving force in the progression of DKD. However, the incidence of DKD is rising despite strict glycemic and blood pressure control regimens [4]. Therefore, many other mechanisms including prolonged microinflammation, oxidative stress, and autophagy are involved in the pathogenesis of DKD [5, 6]. It would be possible to target these mechanisms with specific drugs to delay disease progression.

Synthetic drugs used to treat DKD are loaded with many side effects [7, 8], therefore, phytochemicals are emerging as alternatives [9]. Because phytochemicals are known as natural modifiers that are relatively safe, less toxic, and can interact with multiple targets to retard disease progression [10]. Hence, some phytochemicals may be potent nephroprotective agents. Resveratrol (RES) is a natural non-flavonoid polyphenolic compound isolated from natural plants such as grapes, peanuts, blueberries, bilberries, cranberries, and certain medicinal herbs [8]. RES exhibits diverse pharmacological actions such as cardioprotective, nephroprotective, hepatoprotective, neuroprotective, anti-oxidant, anti-inflammatory, anti-osteoporosis, anti-diabetic, anti-obesity, anti-atherosclerosis, anti-aging, and anti-tumor properties through regulating a broad spectrum of molecular targets and signaling pathways [11, 12]. RES has been widely available as an over-the-counter nutritional supplement with an array of alleged salutary effects [11]. Interestingly, data reporting the use of RES against DKD have been collected from preliminary clinical investigations [13, 14]. As revealed by a previous study, oral supplementation of RES (10 mg/day) for 4 weeks obviously improved insulin resistance and renal function in patients with diabetes [14]. Meanwhile, the urine albumin/creatinine ratio was remarkably reduced in diabetes patients who were treated with RES (500 mg/day) for 90 days, suggesting that RES may be protective against DKD by reducing urinary albumin excretion [13]. However, in-depth preclinical mechanistic studies are currently limited. In addition, details of targets and the signaling pathways through which RES exerts its effects against DKD remain to be further explored.

With the rapid development of bioinformatics, network pharmacology based on big databases has emerged as a powerful tool to characterize the action mechanisms of complicated drug systems in detail, from the molecular level to the pathway level [15, 16]. And network pharmacology is well suited for analyzing multi-targeted agents [17, 18] because it integrates multi-disciplinary technologies, such as systems biology, poly-pharmacology, molecular network data, bioinformatics, and computer simulation. There have been many studies using network pharmacology approaches to reveal the mechanisms of drug actions on diseases. At the same time, this method has emerged as a promising approach to accelerate drug research and development [17]. However, there have been no systematic network pharmacology studies on RES against DKD.

As such, the purpose of this study was designed to use the network pharmacology and molecular docking approach to reveal potential therapeutic targets, biological functions, and the signaling pathways of RES against DKD. Multiple lines of evidence indicate that high glucose (HG)-induced podocyte injury and loss is a critical factor in the development of DKD [19, 20]. Decreased expression levels of podocyte markers, such as nephrin and WT1, have been recognized as an early hallmark of podocyte injury and dysfunction [19]. Therefore, to verify the reliability of the computer simulation results, we also validated the effect of RES on target proteins in the HG-induced podocyte injury model [19, 21]. The present study may provide new insights and scientific evidence for RES as a dietary supplement intervention in DKD.

**Fig. 1:**
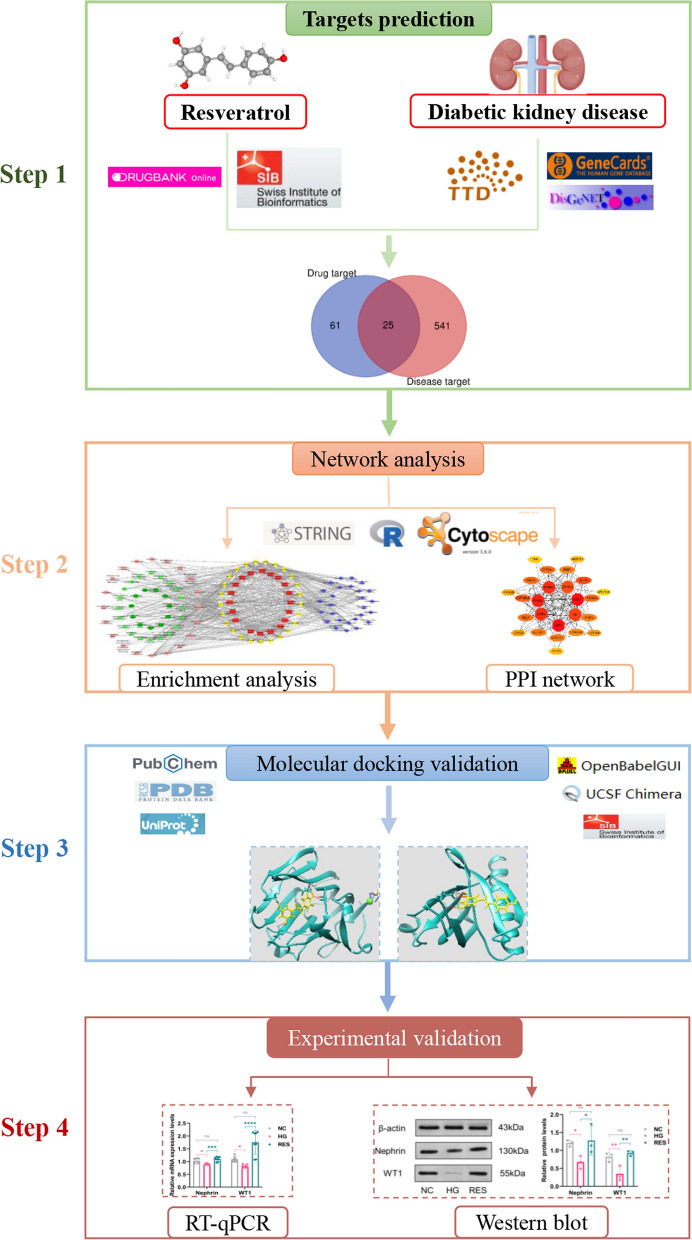
Flowchart of a network pharmacology-based approach to investigate the pharmacologic targets of resveratrol in the treatment of diabetic kidney disease

The flowchart of the present study is illustrated in Fig. 1.

## Materials and methods

### Drug targets of RES

DrugBank is a comprehensive, freely available web-based bioinformatics and cheminformatics database containing detailed drug data and comprehensive information on drug targets, drug actions, and drug interactions [22]. The most significant advantage of DrugBank is its extensive and comprehensive data on drug targets. And these targets are experimentally validated and regularly updated. The SwissTargetPrediction Database is used to predict the most probable protein targets of small molecules based on the similarity of the two-dimensional and three-dimensional chemical structures of compounds [23]. The advantage of the SwissTargetPrediction database is that it can predict the targets of small molecule drugs based on the similarity principle and improve the accuracy based on the prediction score. Drug targets of RES were searched and collected from the Drugbank (accessed in September 2022) and the SwissTargetPrediction Databases (accessed in September 2022). To increase the accuracy of the predictions, data from the above databases were merged and duplicates were removed.

### Therapeutic targets for DKD

DisGeNET covers the most exhaustive and publicly available catalogues of genes and genomic variants associated with diseases [24]. Its advantage lies in its ability to screen for disease-related targets based on gene-disease associations (GDAs) score. Genecards is a comprehensive, authoritative compendium of annotative information about genes [25]. The strength of the Genecards database lies in its ability to find gene-associated diseases by comprehensively calculating the relevance score of diseases and genes. Therapeutic Target Database (TTD) is the first online database in the world that provides drug target information free of charge [26]. The therapeutic targets of agents in the TTD are validated by clinical evidence or patent or literature reports. The merged and de-duplicated targets from the DisGeNET (accessed in September 2022, GDAs score > 0.06) [24], Genecards (accessed in September 2022, Relevance score > 10) [25], and TTD (accessed in September 2022) databases were selected as disease targets of DKD.

The online tool Draw Venn Diagram (http://bioinformatics.psb.ugent.be/webtools/Venn/) was used to identify potential therapeutic targets for RES against DKD. The functional classification of therapeutic targets was performed by Panther database (accessed in September 2022) [27].

### Construction of the protein–protein interaction (PPI) network

The therapeutic targets for RES against DKD were uploaded to the online tool String database [28] with a confidence score > 0.40. Subsequently, the PPI network was visualized by CytoHubba, a plugin in Cytoscape software (version 3.6.0) to calculate the degree of each protein node.

### Construction of the network of diseases-targets-functional annotations-signaling pathways

Gene ontology (GO) functional enrichment analysis, Kyoto Encyclopedia of Genes Genomics (KEGG) pathway analysis, and disease association analysis were performed using the DAVID database (accessed in October 2022) [29]. The therapeutic targets for RES against DKD were uploaded to DAVID. The DISGENET and GAD_DISEASE databases were used to perform disease association analysis (diseases associated with diabetes, kidney diseases, and cardiovascular diseases with a *P* < 0.05 in top 20 Enrichment Score were selected for further analysis). The KEGG_PATHWAY analysis was utilized for pathway enrichment analysis. The GO functional enrichment analysis was performed using GOTERM_BP_DIRECT, GOTERM_CC_DIRECT, and GOTERM_MF_DIRECT. The top 20 enriched KEGG and GO terms with *P* < 0.05 were selected for further analysis. Finally, the diseases-targets-functional annotations-signaling pathways network was visualized using Cytoscape software.

### Molecular docking validation of the binding capacity between RES and targets

The 3-dimensional structure of RES was downloaded from PubChem database [30] and transformed into Mol2 format using Open Babel software [31]. The hydrogen atoms were added using UCSF Chimera software [32]. The template structures of target proteins were obtained from the UniProt database [33] and RCSB Protein Data Bank [34]. All the above databases were accessed in November 2022. The atom charges and hydrogen atoms were added to the protein and the solvents were removed with the UCSF Chimera. The molecular docking of RES and target proteins was performed using the SwissDock webserver (accessed in December 2022) [35] and visualized by UCSF Chimera.

### Cell culture

The mouse podocyte clone-5 (MPC-5) cell lines were purchased from the American type culture collection. MPC-5 cells were cultured in Dulbecco’s modified Eagle’s medium (DMEM, Cytiva, Marlborough, Massachusetts, United States, Cat# SH30022.01) containing 10% fetal bovine serum (Biological Industries, Kibbutz Beit Haemek, Israel, Cat# 04-001-1A), 100 U/ml penicillin, 100 μg/ml streptomycin, and 0.25 μg/ml amphotericin B (Zhong Qiao Xin Zhou Biotechnology Co., Ltd., Shanghai, China, Cat#CSP007) in a 37℃, 5% CO_2_ incubator (Panasonic MCO-18AC, Osaka, Japan). The culture medium was changed every 2 days. When the cells were in good condition and fused to about 80%, they were divided into the normal control group (NC group, 30 mM mannitol), high glucose group (HG group, 30 mM glucose) [19, 21], and RES-treated group (30 mM glucose plus 5 μM RES) [36] to continue the culture process. Mannitol (Cat# SM8120, purity ≥ 98%) and glucose (Cat# G8150, purity ≥ 99.8%) powder was purchased from Solarbio Technology Co., Ltd. (Beijing, China) and dissolved in DMEM. RES was purchased from Aladdin Industrial Company (Shanghai, China, Cat# R107315, purity 99%) and dissolved in dimethyl sulfoxide (Sigma, Saint Louis, Missouri, United States, Cat# D8418). The cells were collected 72 h after the treatment.

### Reverse transcription-quantitative polymerase chain reaction (RT-qPCR)

The total RNA was extracted from the MPC-5 cells in NC, HG, and RES groups by using SteadyPure Universal RNA Extraction Kit II (Accurate biotechnology Co., Ltd., Hunan, China, Cat# AG21022) according to the manufacturer’s instructions. RNA concentrations were measured on a Nano-300 Micro-Spectrophotometer (Allsheng Instruments Co., Ltd., Hangzhou, China). If D(λ)260/D(λ)280 was 1.8–2.0, RNA was stored in a − 80 ℃ refrigerator for later use. A total of 1 μg RNA was taken to synthesize complementary DNA by using Evo M-MLV RT Premix for qPCR AG (Accurate biotechnology Co., Ltd., Hunan, China, Cat# AG11706). The reverse transcription was performed at 37 ℃ for 15 min, 85 ℃ for 5 s, and 4 ℃ on a Bio-Rad S1000TM Thermal Cycler (Bio-Rad Laboratories, Hercules, California, United States). All primers for PCR amplification were designed using NCBI Primer-BLAST and purchased from Shanghai Sangon Biotechnology. The primer sequences are shown in Table 1. RT-qPCR was carried out in a Bio-Rad CFX-96 Real-time PCR system (Bio-Rad Laboratories, Hercules, California, United States) using the SYBR^®^ Green Premix Pro Taq HS qPCR Kit (Accurate biotechnology Co., Ltd., Hunan, China, Cat# AG11701). PCR amplification was performed with an initial denaturation at 95 ℃ for 30 s, followed by 40 cycles of 95℃ for 5 s and annealing at 60 ℃ for 30 s. Expression of the β-actin gene was assayed simultaneously with samples as an internal control. Relative gene expression was determined by the 2^−ΔΔCT^ method. All samples were performed three times in parallel. The specificity of each primer pair was verified both by in silico analysis (NCBI Primer-BLAST) and melting curve analysis after qPCR amplification.

**Table 1:** Sequences of PCR primers

Gene symbol	Accession number	Forward primer (5′–3′)	Reverse primer (5′–3′)	Expected amplicon size (base pair)
β-actin	NM_007393	TATGCTCTCCCTCACGCCATCC	GTCACGCACGATTTCCCTCTCAG	129
Nephrin	NM_019459	CTCCACGGTTAGCACAGCAGAAG	GCTTGGCGATATGACACCTCTTCC	102
WT1	NM_144783	CAGTGAAATGGACAGAAGGGCAGAG	ATACACGCCGCACATCCTGAATG	141
PPARA	NM_011144	CTTCACGATGCTGTCCTCCTTGATG	GATGTCACAGAACGGCTTCCTCAG	112
ESR1	NM_007956	CCTGGCTGGAGATTCTGATGATTGG	TCCACCATGCCTTCCACACATTTAC	124
SLC2A1	NM_011400	GATGAAAGAAGAGGGTCGGCAGATG	CAGCACCACAGCGATGAGGATG	106
SHBG	NM_011367	CTTCTGCTTCCTTCTGCCTGAGTG	CTAGTGGGAGGTGCGGGTATCG	140
AR	NM_013476	GGCAGCAGTGAAGCAGGTAGC	CAGAGCCAGCGGAAAGTTGTAGTAG	129
AKR1B1	NM_009658	AAGCCTGAAGATCCGTCTCTCCTG	CGCACTGGTGTCACAGACTTGG	143
PPARG	NM_011146	GCCAAGGTGCTCCAGAAGATGAC	GTGAAGGCTCATGTCTGTCTCTGTC	102
IGF1R	NM_010513	ATGCCAACAAGTTCGTCCACAGAG	TCCGTCTCGTAGATGTCTCGTGTC	112
RELA	NM_009045	CCAGACCAACAACAACCCCTTCC	AAGCAGAGCCGCACAGCATTC	81
PIK3CA	NM_008839	GCACAAGAGTACACCAAGACCAGAG	GCATTCCAGAGCCAAGCATCATTG	130
MMP9	NM_013599	CGCCACCACAGCCAACTATGAC	CTGCTTGCCCAGGAAGACGAAG	130
AKT1	NM_009652	TCAGGATGTGGATCAGCGAGAGTC	AGGCAGCGGATGATAAAGGTGTTG	108
INSR	NM_010568	ATCCGCCGCTCCTATGCTCTG	GAGTTGCCTCAGGTTCTGGTTGTC	126
MMP2	NM_008610	ACCATGCGGAAGCCAAGATGTG	AGGGTCCAGGTCAGGTGTGTAAC	126
TTR	NM_013697	ACACTTGGCATTTCCCCGTTCC	GCGATGGTGTAGTGGCGATGG	83

### Western Blot

The protein in each group was extracted by using RIPA lysis buffer (Epizyme, Shanghai, China, Cat# PC101) containing 1 mM phosphatase inhibitors (Epizyme, Shanghai, China, Cat# GRF102), and 1 mM protease inhibitors (Epizyme, Shanghai, China, Cat# GRF101). Cell lysates were centrifuged at 10,625 × *g* and 4℃ for 15 min. Then the total protein level of the supernatant was quantified by using the bicinchoninic acid protein assay kit (NCM biotechnology Co., Ltd., Suzhou, China, Cat# WB6501). Supernatants were then mixed with 5 × protein sample loading buffer (Epizyme, Shanghai, China, Cat# LT101), boiled for 10 min, and stored at – 20 ℃ for later use. Equal amounts of protein (40 μg/lane) from each sample were loaded on 10% SDS-PAGE gel and then transferred to the 0.45 μm polyvinylidene difluoride membrane. The gel blocking was performed with 5% nonfat dry milk (BioFroxx, Berlin, Germany, Cat# 1172GR500) in Tris-buffered saline containing 0.1% Tween-20 (TBST) (BioFroxx, Berlin, Germany, Cat# 1247ML100) at room temperature for 2 h, followed by incubation with primary antibodies overnight at 4 ℃. Primary antibodies β-actin (Cat#AF7018, diluted to 1:1000), Nephrin (Cat#AF7951, diluted to 1:2000) were purchased from Affinity Biosciences (Jiangsu, China) and WT-1 (Cat#380,948, diluted to 1:1000) was purchased from Zen-Bioscience (Chengdu, China). After washing with 1 × TBST solution for three times (10 min each), membranes were incubated with HRP-conjugated goat anti-rabbit secondary antibody (Epizyme, Shanghai, China, Cat# LF102, diluted to 1:7000) for 1.5 h at room temperature. After washing three times with 1 × TBST, chemiluminescence detection reagents (Epizyme, Shanghai, China, Cat# SQ201) were used to visualize the bands via the Amersham Imager 600 (General Electric Company, Boston, Massachusetts, United States). Densitometric analysis of the bands was performed with Image J software and relative protein expression was determined by normalizing to β-actin.

### Statistical analysis

Data from western blot and RT-qPCR experiments were plotted and analyzed by using GraphPad Prism 9.0 and Statistical software SPSS 26.0 software. The normality of the data was tested by the Shapiro–Wilk test and *P* > 0.05 was considered as normal distribution. Homogeneity of variance was tested using Levene’s test with *P* > 0.05 considered homogenous. For data that met normal distribution assumptions and equal variances, statistical analyses were performed through one-way analysis of variance (ANOVA) and least significant difference (LSD) post hoc test for multiple comparisons. Otherwise, the multiple sample rank-sum tests were performed using the Kruskal–Wallis test and *P*-values for multiple comparisons were adjusted using Bonferroni correction. A value of *P* < 0.05 was considered statistically significant.

## Results

### Identification of RES drug targets

There were 26 and 69 RES drug targets in Drugbank and SwissTargetPrediction databases respectively. We combined these drug targets and removed 9 duplicates. Finally, 86 drug targets were selected for further analysis.

### Identification of disease targets

In the DisGeNET, Genecards, and TTD databases, 224, 437, and 22 targets were associated with DKD, respectively. After removing redundant targets, 566 DKD-related targets were obtained.

### Identification of therapeutic targets for RES against DKD

**Fig. 2:**
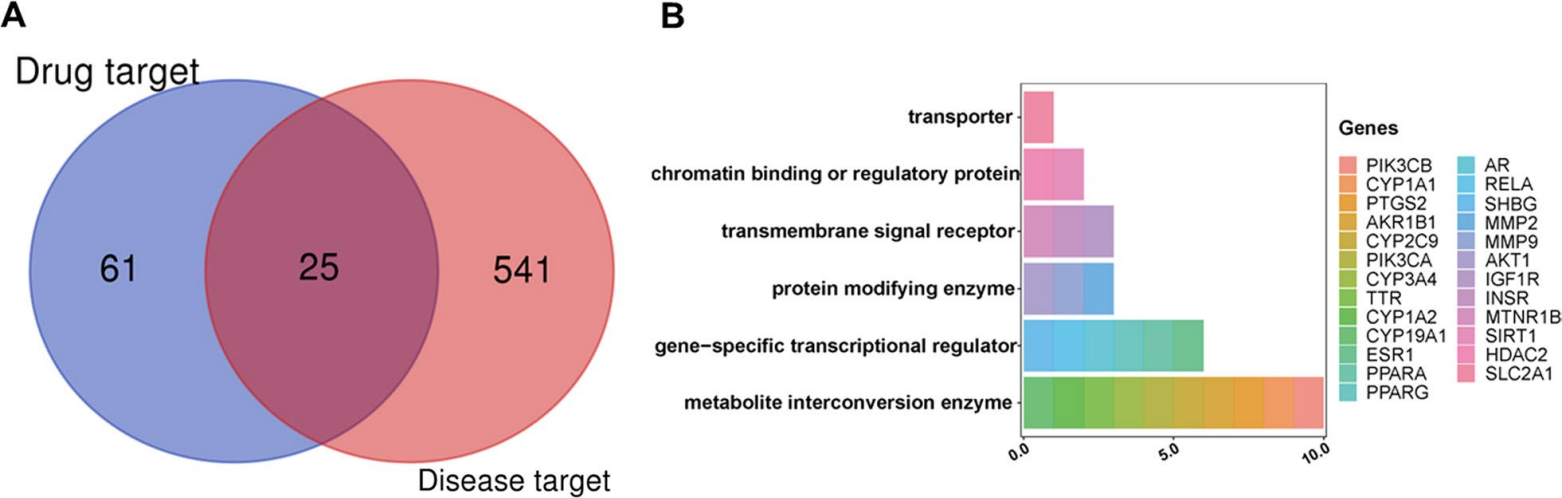
Identification of therapeutic targets for RES against DKD. **A** Venn diagram of potential therapeutic targets. **B** Classification of the identified targets. By taking the intersection of 86 drug targets and 566 DKD-related targets, 25 therapeutic targets for RES against DKD can be obtained and classified into 6 functional categories. RES: resveratrol, DKD: diabetic kidney disease

The above RES drug targets were intersected with DKD targets to obtain potential therapeutic targets for RES against DKD. Finally, a total of 25 targets were identified (Fig. 2A). And these targets can be classified into 6 functional categories including metabolite interconversion enzyme (PIK3CA, PIK3CB, CYP1A1, CYP1A2, CYP2C9, CYP3A4, CYP19A1, TTR, PTGS2, and AKR1B1), gene-specific transcriptional regulator (ESR1, AR, SHBG, PPARA, PPARG, and RELA), protein modifying enzyme (AKT1, MMP9, and MMP2), transmembrane signal receptor (IGF1R, INSR, and MTNR1B), chromatin-binding or regulatory protein (SIRT1 and HDAC2), and transporter (SLC2A1) (Fig. 2B).

### 3.4. PPI network construction

**Fig. 3:**
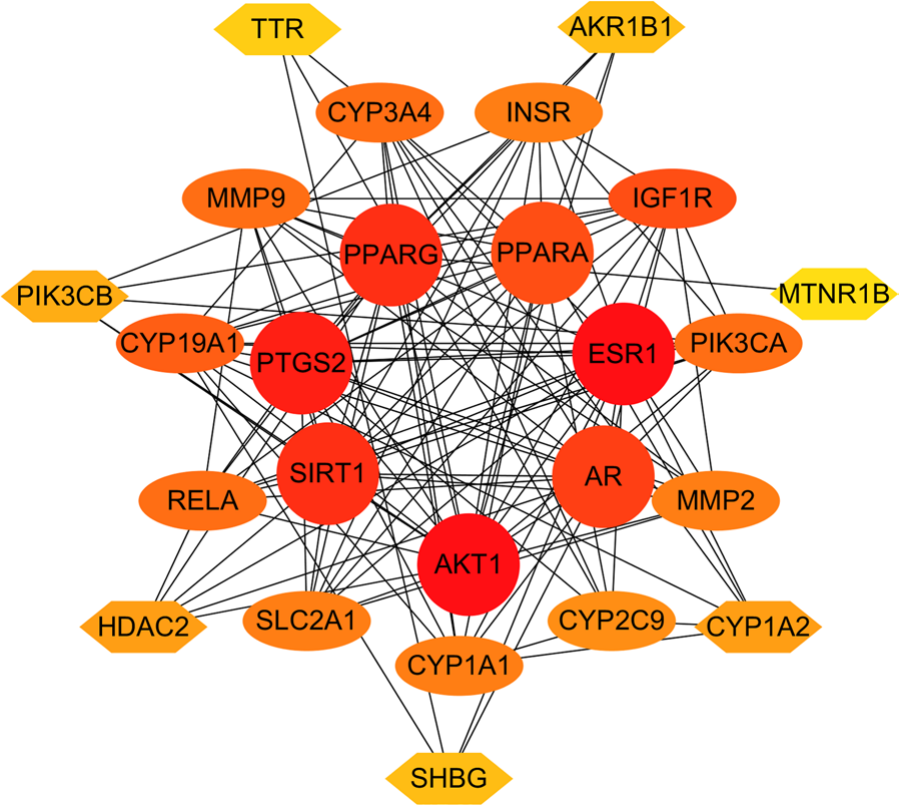
Protein–protein interaction network of RES targets against DKD. The color of the nodes reflects the degree of connectivity (the redder color indicates a higher degree). RES: resveratrol, DKD: diabetic kidney disease

The identified 25 therapeutic targets for RES against DKD were used to construct the PPI network. A total of 25 nodes and 123 edges were included in the PPI network, with an average node degree of 9.84 (Fig. 3). The ranking of the therapeutic targets based on the weighted degree was AKT1, ESR1, PTGS2, PPARG, SIRT1, AR, PPARA, IGF1R, CYP19A1, PIK3CA, CYP3A4, MMP9, RELA, MMP2, INSR, SLC2A1, CYP1A1, CYP2C9, CYP1A2, HDAC2, PIK3CB, AKR1B1, SHBG, TTR, and MTNR1B.

### Diseases-targets-functional annotations-signaling pathways network

**Fig. 4:**
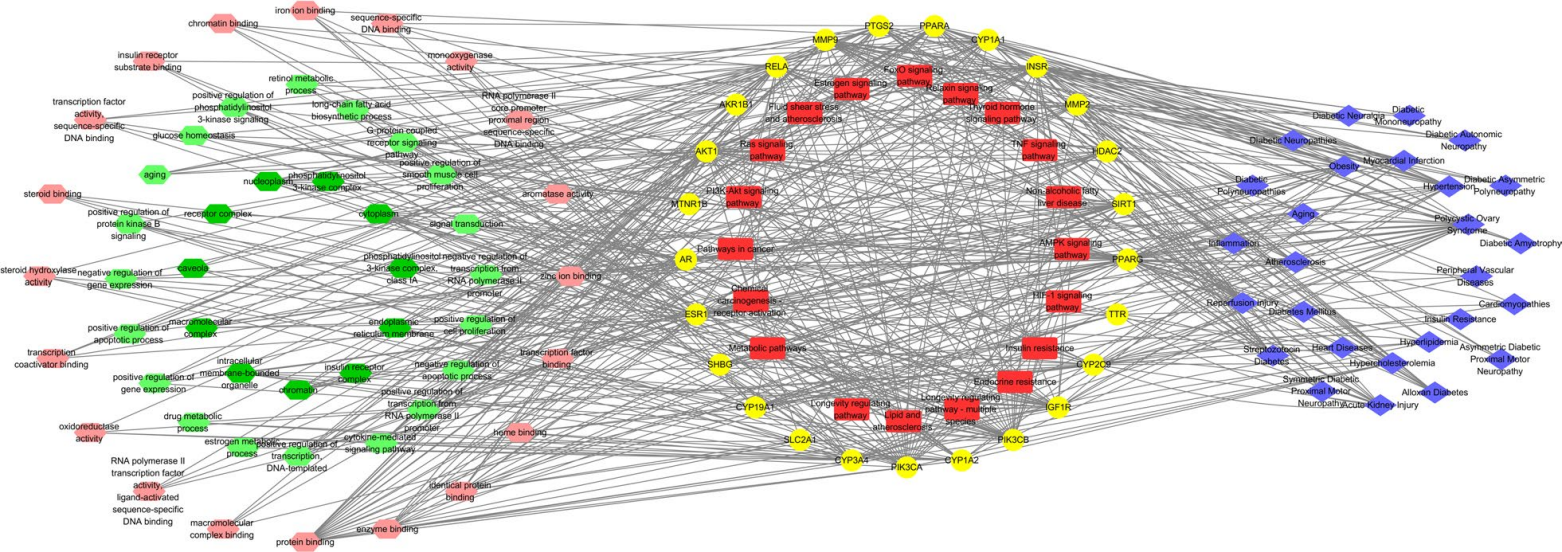
Diseases-targets-functional annotations-signaling pathways network. GO terms are shown on the left side. The dark green hexagon represents the cellular component. The light green hexagon represents the biological process. The pink hexagon represents the molecular function. The central yellow circle represents RES targets against DKD. The central red round rectangle represents the enriched KEGG pathway. Diseases enrichment analyses are indicated by blue diamonds on the right side. GO: Gene ontology, RES: resveratrol, DKD: diabetic kidney disease, KEGG: Kyoto Encyclopedia of Genes Genomics

GO, KEGG, and disease enrichment analysis of RES targets against DKD were performed in DAVID database. A total of 11 cellular component terms were enriched. And the top 20 enriched biological processes, molecular functions, and KEGG pathways are shown in Fig. 4. After removing the repetitions, a total of 27 diseases were enriched in DISGENET and GAD_DISEASE databases.

### Validation of binding capacity between RES and therapeutic targets by molecular docking

**Fig. 5:**
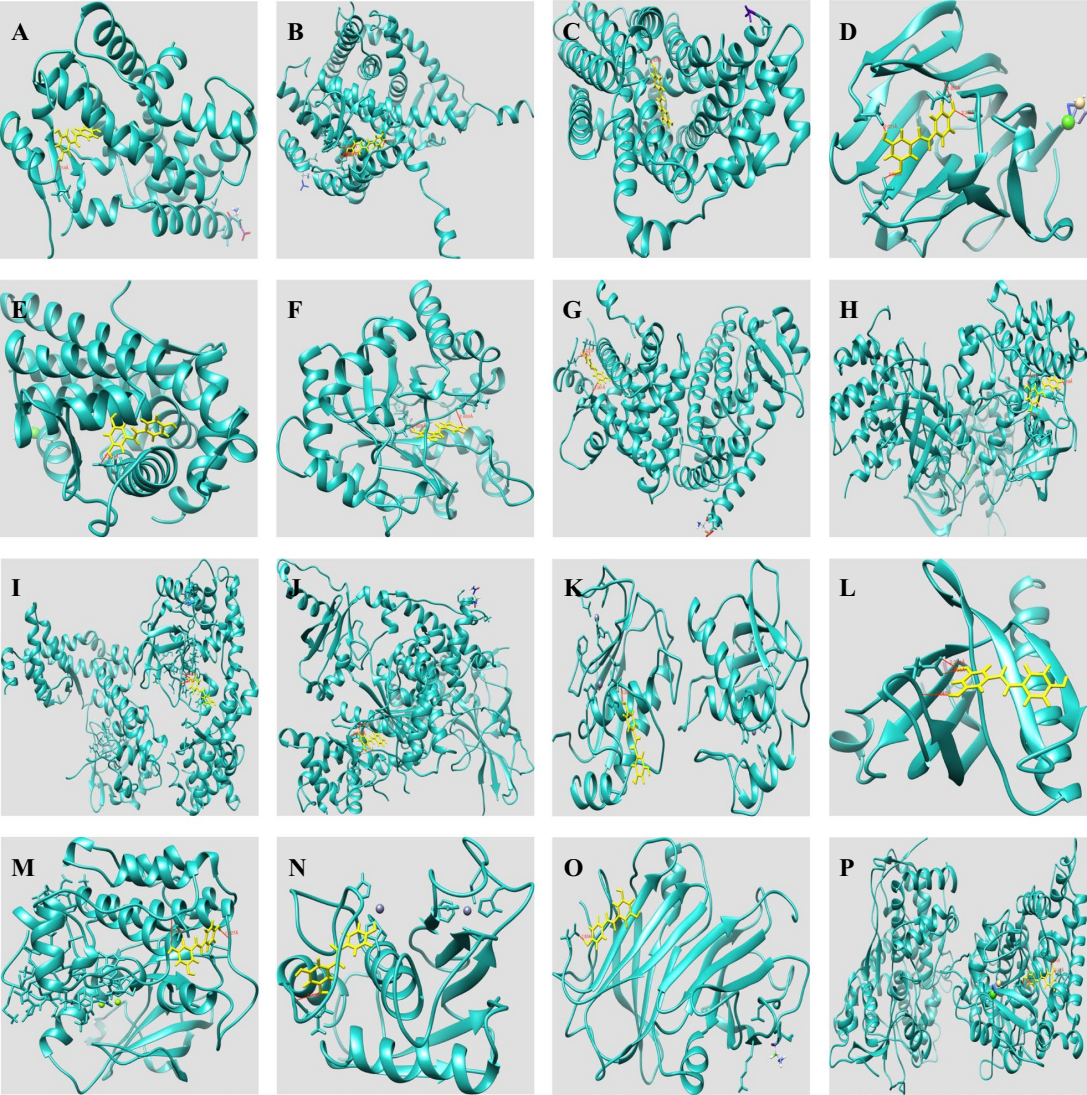
The 3-dimensional map of the binding sites between RES and target proteins **A** PPARA, **B** ESR1, **C** SLC2A1, **D** SHBG, **E** AR, **F** AKR1B1, **G** PPARG, **H** IGF1R, **I** RELA, **J** PIK3CA, **K** MMP9, **L** AKT1, **M** INSR, **N** MMP2, **O** TTR, **P** CYP2C9. RES is shown in yellow. Target proteins are displayed as cyan. The places where RES and the target proteins are connected represent specific docking sites between RES and target proteins. RES: resveratrol

The binding capacity of RES and target proteins were predicted by SwissDock webserver. The docking models with the lowest binding energy (Delta G) were recorded. Docking sites of RES with PTGS2, SIRT1, CYP1A1, HDAC2, CYP19A1, CYP3A4, CYP1A2, and MTNR1B were not obtained from the SwissDock database. The other molecular docking results of RES with target proteins are shown in Table 2. And the results showed that RES had the strongest and most stable binding affinity toward PPARA, followed by ESR1, SLC2A1, SHBG, AR, AKR1B1, PPARG, IGF1R, RELA, PIK3CA, MMP9, AKT1, INSR, MMP2, TTR, and CYP2C9. The specific docking sites of RES and target proteins can be seen in Fig. 5.

**Table 2:** Molecular docking results of RES with target proteins

Drug	Targets	PDB ID	Energy (kcal/mol)	FullFitness (kcal/mol)	Estimated Delta G (kcal/mol)	Delta Gvdw
RES	PPARA	1I7G	− 6.74225	− 1608.3	− 7.98322	− 47.0982
RES	ESR1	1A52	− 4.39569	− 2430.87	− 7.97234	− 46.6933
RES	SLC2A1	6THA	− 8.32298	− 1303.2	− 7.72539	− 45.941
RES	SHBG	6PYB	− 4.02258	− 1018.55	− 7.72529	− 44.1123
RES	AR	5CJ6	− 7.58022	− 1321.28	− 7.50935	− 41.8967
RES	AKR1B1	4PRT	− 9.30686	− 1535.5	− 7.50572	− 44.0213
RES	PPARG	6C1I	− 5.15499	− 3211.41	− 7.43626	− 38.423
RES	IGF1R	1JQH	− 2.36975	− 5357.23	− 7.18328	− 40.8937
RES	RELA	3RC0	− 2.15755	− 4707.42	− 7.14368	− 35.1925
RES	PIK3CA	6GVH	− 4.2462	− 4944.28	− 7.06001	− 35.1123
RES	MMP9	1GKC	− 2.01194	− 1975.08	− 6.97222	− 36.4449
RES	AKT1	1H10	2.16551	− 1057.25	− 6.89505	− 2151.35
RES	INSR	1IR3	− 0.910791	− 2075.83	− 6.87624	− 32.0733
RES	MMP2	1HOV	− 1.63272	− 1190.34	− 6.7931	− 32.056
RES	TTR	1BZ8	5.40908	− 1487.5	− 6.58621	− 31.7126
RES	CYP2C9	1OG2	− 1.04103	− 4625.68	− 6.30214	− 37.527

### Effect of RES on HG-induced podocyte injury model

**Fig. 6:**
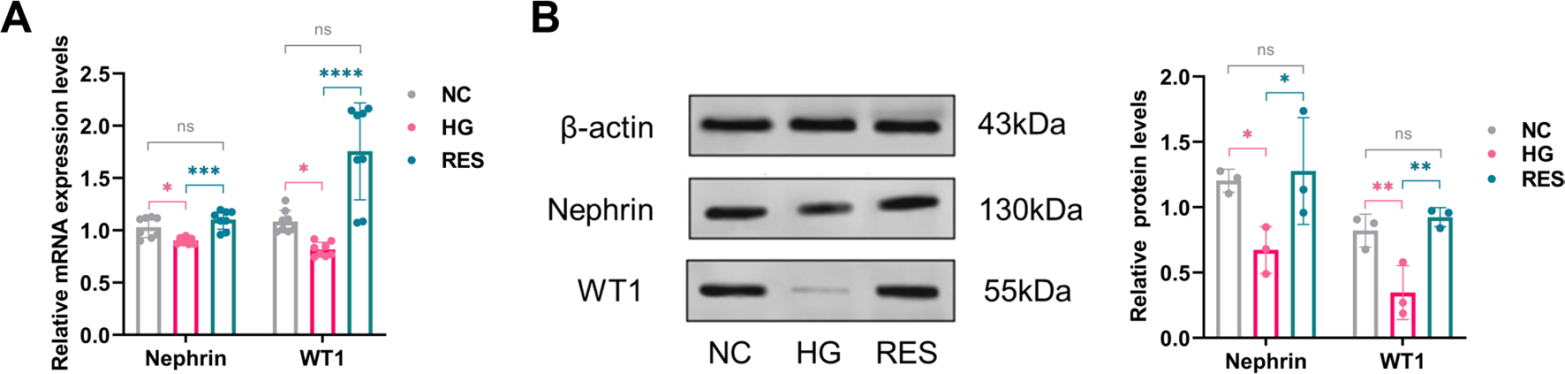
Effect of RES on HG-induced podocyte injury model. The relative **A** mRNA and **B** protein levels of nephrin and WT1 are downregulated in the HG group compared with those in the NC group. And treatment with RES increase the expression levels of nephrin and WT1. NC: normal control, HG: high glucose, RES: resveratrol, ns: *P* > 0.05, *P < 0.05, **P < 0.01, ***P < 0.001, ****P < 0.0001

Murine podocytes were treated with NC, HG, HG plus 5 μM RES for 72 hours. The mRNA and protein expression levels of podocyte markers, including nephrin and WT1, were analyzed by RT-qPCR and western blot. The relative mRNA and protein levels of nephrin and WT1 were significantly downregulated in the HG group compared with those in the NC group (Fig. 6). It indicates that an in vitro HG-induced podocyte injury model had been successfully constructed. Treatment with RES strongly increased the mRNA and protein expression of nephrin and WT1. And there was no statistically significant difference in the expression of nephrin and WT1 between the RES and NC groups (Fig. 6).

### Effect of RES on target proteins

**Fig. 7:**
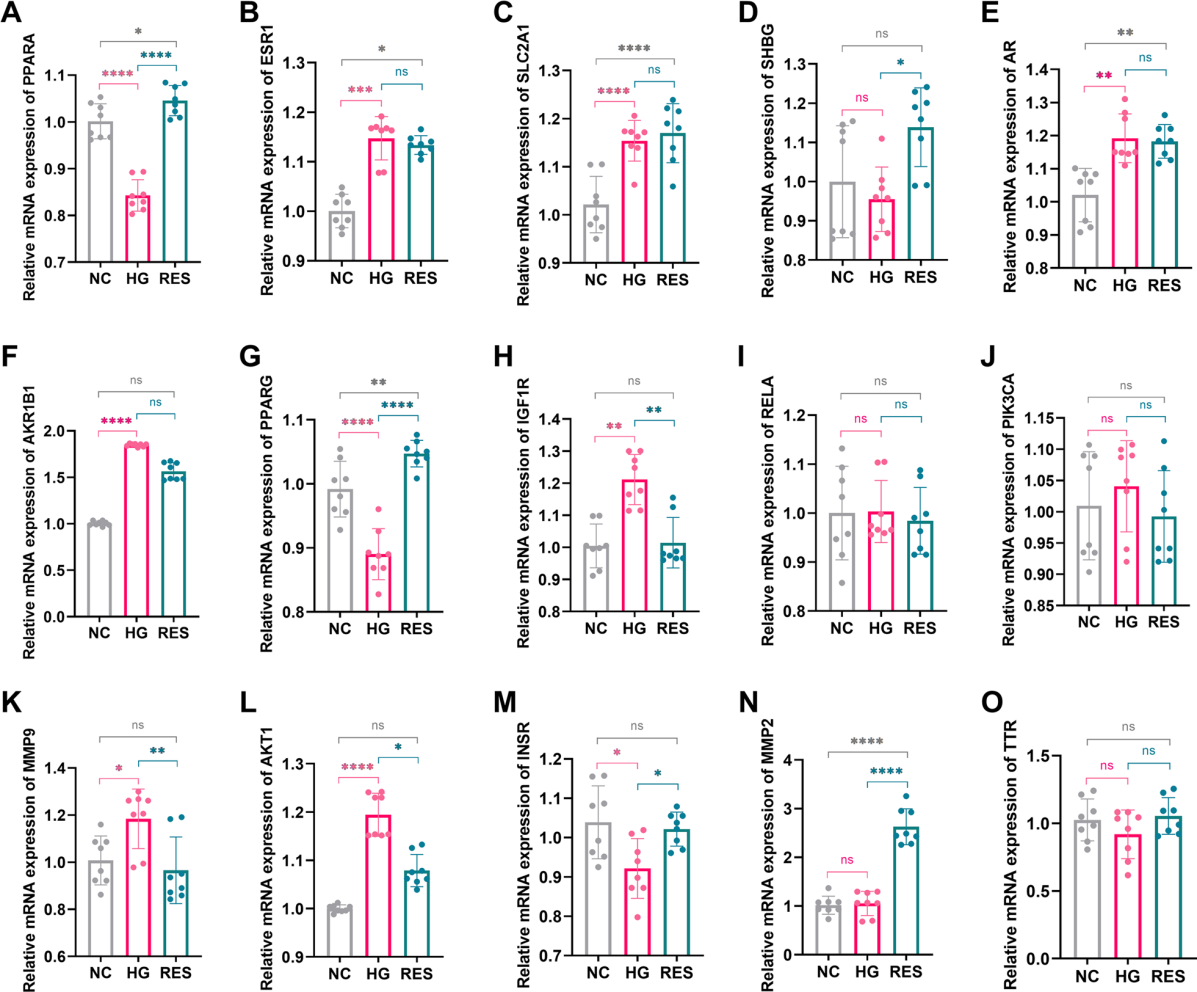
Effect of RES on target proteins. The mRNA expression levels of ESR1 (**B**), SLC2A1 (**C**), AR (**E**), AKR1B1 (**F**), IGF1R (**H**), MMP9 (**K**), and AKT1 (**L**) in the HG group are significantly increased, whereas PPARA (**A**), PPARG (**G**), and INSR (**M**) levels are significantly decreased in the HG group compared with the NC group. There are no significant differences in the expression of the SHBG (**D**), RELA (**I**), PIK3CA (**J**), MMP2 (**N**), and TTR (**O**) between HG and NC groups. RES treatment is able to reverse the abnormal gene expression of PPARA, SHBG, AKR1B1, PPARG, IGF1R, MMP9, AKT1, and INSR in the HG group. NC: normal control, HG: high glucose, RES: resveratrol, ns: *P* > 0.05, *P < 0.05, **P < 0.01, ***P < 0.001, ****P < 0.0001

The mRNA expression levels of target proteins in the NC, HG, and RES-treated groups were measured by RT-qPCR. The PCR results (Fig. 7) showed that the mRNA expression levels of ESR1, SLC2A1, AR, AKR1B1, IGF1R, MMP9, and AKT1 in the HG group were significantly higher than those in the NC group. The expression levels of PPARA, PPARG, and INSR were significantly lower in the HG group compared with the NC group. RES treatment at 5 μM reversed the abnormal expression of PPARA, SHBG, AKR1B1, PPARG, IGF1R, MMP9, AKT1, and INSR in the HG group.

## Discussion

With the rapid development of the economy and society, the prevalence of DKD is gradually increasing worldwide which imposes a heavy economic and social burden. Our previous bioinformatics analysis found that RES can reverse gene expression abnormalities in DKD patients’ glomerulus [37]. Moreover, as a natural active ingredient, RES has the characteristics of being non-toxic and harmless [38]. The present study explored the potential therapeutic targets for RES against DKD. We found that the identified targets were also associated with other kidney diseases, diabetic and cardiovascular complications.

To further explore the specific binding sites where RES plays the above roles, we performed molecular docking validation. Generally, results of molecular docking are mainly evaluated by minimum binding free energy (Delta G) [39]. The lower the binding energy is, the less energy is required for the drug molecule to bind with the target, and it indicates a greater binding possibility and stability [40, 41]. Through molecular docking and in vitro experiments, we found that RES can ameliorate HG-induced podocyte injury by acting on PPARA, SHBG, AKR1B1, PPARG, IGF1R, MMP9, AKT1, and INSR.

Many studies have proved the benefits of PPARA activators. A previous study has indicated that RES treatment can increase the expression level of PPARA and restore normoglycemia and insulin secretion in diabetic rats [42]. A bioaffinity chromatographic study showed that RES has a direct interaction with the immobilized ligand-binding domains of PPARA and PPARG [43]. Another study also showed that RES can prevent DKD by ameliorating lipotoxicity, oxidative stress, apoptosis, and endothelial dysfunction through AMPK-SIRT1-PPARA axis [44]. Similar to PPARA, PPARG is also a transcription factor activated by ligands. RES can activate PPARG directly [45] or suppress the inhibitory effects of NF-κB on PPARG [46]. A previous study showed that RES can ameliorate podocyte damage by attenuating mitochondrial oxidative stress and apoptosis via activation of SIRT1/PPARG coactivator lα [47]. Thus, RES may play an important role in alleviating DKD by directly targeting or indirectly activating PPARA and PPARG.

SHBG is a plasma glycoprotein that regulates the action of steroid hormones including androgens and estradiol [48]. Previous researches have shown that low serum SHBG is related to obesity, insulin resistance, compensatory hyperinsulinemia, and is a biomarker for abnormal glucose and lipid metabolism, type 2 diabetes, and cardiovascular diseases [49, 50]. And it has also been corroborated that RES can increase hepatic SHBG production [50] and increase circulating levels of SHBG [51]. Our current study proved that RES can directly target SHBG to increase the expression level of SHBG in DKD status. This may be because RES can bind to the DR-1 element which is located upstream of the transcription start site of the SHBG proximal promoter, thus improving the expression level of SHBG mRNA [50]. Therefore, RES may improve glucose metabolism and reduce the risk of cardiovascular diseases in DKD patients by targeting SHBG.

AKR1B1 is one of a subfamily members of aldo‐keto reductase family 1 (AKR1) which plays an important role in glucose metabolism [52, 53]. The overexpression of AKR1B1 is closely associated with inflammatory mediators, and diabetic eye disease [54]. Inhibition of AKR1B1 has a supportive role in the reduction of superoxides and toxic substances [52]. AKR1B1 inhibitors, such as tolrestat and epalrestat, have been used clinically to treat diabetic complications [54]. Similarly, IGF1R plays a critical role in the development of DKD by regulating inflammation, cell growth, oxidative stress, and promoting cell proliferation and fibrosis [55]. It has been proved that the expression of IGF-1R is significantly elevated in the kidney of diabetic rats [56]. Therefore, elevated IGF1R may contribute to the development of DKD. And IGF1R inhibitor could alleviate glomerulomegaly, inflammatory infiltration, and tubulointerstitial fibrosis to slow down the development of DKD [55, 56]. Our current study showed that the expression levels of AKR1B1 and IGF1R were significantly upregulated in the HG group. However, there was no statistically significant difference in the expression levels of AKR1B1 and IGF1R between the RES intervention group and the NC group. It has been indicated that sp1 is a critical mediator of IGF1R transcription because it can upregulate IGF1R promoter activity [57]. Besides that, another study showed that p53 can decrease IGF1R promoter activity [58]. RES can suppress sp1 and activate p53 to inhibit the transcription of IGF1R [57]. Therefore, RES was able to act as a potential inhibitor of AKR1B1 and IGF1R to delay DKD progression.

MMP9 and MMP2 are members of the matrix metalloproteinase family that provide homeostasis between the synthesis and degradation of the extracellular matrix to maintain the structural and functional integrity of the glomerulus [59]. It has been proved that MMP9 increased significantly in the urine, serum, and renal tissues of DKD [59]. The overexpression of MMP9 can induce podocyte dedifferentiation, and interrupt the integrity of the podocyte [60]. Meanwhile, upregulation of urinary MMP9 concentrations occurred earlier than the onset of microalbuminuria, and the serum levels of MMP9 are inversely associated with estimated glomerular filtration rate (eGFR) [59]. Moreover, MMP9 knockout significantly ameliorated albuminuria and prevented the above structural alterations in nephropathy [60]. Therefore, MMP9 is a reliable biomarker for the early diagnosis of DKD. Our current study also confirmed that MMP9 expression was significantly increased in the HG group. And the application of RES was able to reverse this trend. Luciferase reporter assays confirmed that RES dose-dependently decreases the promoter activity of MMP9 [61]. Therefore, RES can directly inhibit the transcriptional activity of MMP9. At the same time, NF-κB and AP-1 are important modulators for MMP9 transcription [61]. The inhibitory effect of RES on MMP9 may also be achieved by suppressing the nuclear transcription factor AP-1 [62] and nuclear translocation of p65 [63]. However, the mechanism by which RES exerts its effects on MMP2 remains unclear and controversial. To date, there has been a study indicating that RES affects neither the expression nor the activity of MMP2 [61]. There is also a study showing that RES can significantly inhibit the activation of MMP2 [64]. On the contrary, there is opposing evidence suggesting that RES can increase the secretion of MMP2 [65]. Our current study showed that RES can increase the expression of MMP2. Therefore, we tend to support the idea that the expression and activity of MMP2 are increased as a compensatory mechanism in the early phase of DKD, while MMP2 expression is reduced in advanced DKD [66].

AKT1, also known as protein kinase B, is a key regulatory kinase that transduces signals through the phosphoinositide 3-kinase (PI3K)-AKT signaling cascade to control cell growth and survival [67]. In the present study, we found that PI3K-AKT signaling pathway is one of the top 20 enriched KEGG pathways. It indicates that the above therapeutic targets play therapeutic effects on DKD through PI3K-AKT signaling pathway. INSR is a high-affinity receptor protein for insulin, which can stimulate glucose transport and glycogen synthesis and also inhibit glycogenolysis [68]. The binding of insulin to INSR can also target PI3K-AKT and insulin signaling pathways to regulate glucose metabolism. Many previous studies have proved that RES can improve renal insulin signaling to ameliorate insulin resistance [69], and provide a neuroprotective [70] and cardioprotective effect [71] by targeting PI3K-AKT pathway. Results from RES-appended affinity column chromatography demonstrated that RES is able to bind tightly to AKT and changes its conformation, thereby inhibiting its activity [72]. Therefore, RES may control the development of diabetic complications by targeting AKT1 and INSR.

Accordingly, RES may be a promising pharmacological agent which comprehensively regulates the above therapeutic targets to counteract the development of diabetic complications including DKD. And RES may be recommended as a dietary supplement for delaying the progression of DKD at an early stage. At the same time, RES intervention may also contribute to delaying the development of other diabetic complications.

The strengths of the current study are that we identified comprehensive therapeutic targets for RES against DKD through network pharmacology analysis which integrated various databases, and validated the identified targets by molecular docking simulations and in vitro experiments. To the best of our knowledge, there have been no published studies available conducting such a comprehensive screening and validation of the therapeutic targets for RES against DKD. These findings comprehensively reveal the potential therapeutic targets for RES against DKD and provide theoretical bases for the application of RES in the treatment of DKD.

However, several limitations also existed in the study. First, we only verified the identified targets through computer simulations and in vitro experiments, without conducting in vivo experiments. Second, we did not compare RES with other hypoglycemic drugs, nor did we conduct a combined intervention to further assess the effect of RES on DKD. Third, the results of the present study didn’t provide a reference for the optimal intervention dose of RES in clinical application. Therefore, further in vitro and in vivo experiments are needed subsequently to confirm the mechanisms and effects of RES against DKD in the presence of other hypoglycemic agents. And additional pharmacological studies are needed to establish the optimum dose and bioavailability of RES to maximize long-term cost-effectiveness.

## Conclusions

Aside from the above limitations, we conclude that therapeutic targets for RES against DKD are screened comprehensively via network pharmacology, bioinformatics analysis, molecular docking simulation, and in vitro cell evaluations. RES treatment can reverse the abnormal gene expression of PPARA, SHBG, AKR1B1, PPARG, IGF1R, MMP9, AKT1, and INSR. These findings comprehensively reveal the potential therapeutic targets for RES against DKD and provide theoretical bases for the application of RES in the treatment of DKD.

## Data Availability

All data analysed during this study are included in the websites mentioned above.

## Abbreviations

DKD: Diabetic kidney disease
RES: Resveratrol
HG: High glucose
GDAs: Gene-disease associations
TTD: Therapeutic Target Database
PPI: Protein–protein interaction
GO: Gene ontology
KEGG: Kyoto Encyclopedia of Genes Genomics
MPC-5: Mouse podocyte clone-5
DMEM: Dulbecco’s modified Eagle’s medium
NC: Normal control
RT-qPCR: Reverse transcription-quantitative polymerase chain reaction
TBST: Tris-buffered saline containing 0.1% Tween-20
AKR1B1: Aldo–keto reductase family 1 member B1
AKT1: AKT serine/threonine kinase 1
AR: Androgen receptor
CYP19A1: Cytochrome P450 family 19 subfamily A member 1
CYP1A1: Cytochrome P450 family 1 subfamily A member 1
CYP1A2: Cytochrome P450 family 1 subfamily A member 2
CYP2C9: Cytochrome P450 family 2 subfamily C member 9
CYP3A4: Cytochrome P450 family 3 subfamily A member 4
ESR1: Estrogen receptor 1
HDAC2: Histone deacetylase 2
IGF1R: Insulin like growth factor 1 receptor
INSR: Insulin receptor
MMP2: Matrix metallopeptidase 2
MMP9: Matrix metallopeptidase 9
MTNR1B: Melatonin receptor 1B
PIK3CA: Phosphatidylinositol-4,5-bisphosphate 3-kinase catalytic subunit alpha
PIK3CB: Phosphatidylinositol-4,5-bisphosphate 3-kinase catalytic subunit beta
PPARA: Peroxisome proliferator activated receptor alpha
PPARG: Peroxisome proliferator activated receptor gamma
PTGS2: Prostaglandin-endoperoxide synthase 2
RELA: RELA proto-oncogene, NF-kB subunit
SHBG: Sex hormone binding globulin
SIRT1: Sirtuin 1
SLC2A1: Solute carrier family 2 member 1
TTR: Transthyretin
eGFR: Estimated glomerular filtration rate
PI3K: Phosphoinositide 3-kinase
